# Revisiting 15 000 hours: towards sustainable school systems for mental health, well-being and learning

**DOI:** 10.1192/bjo.2025.10058

**Published:** 2025-07-21

**Authors:** Susan M. Sawyer, Monika Raniti, Rohan Borschmann

**Affiliations:** Centre for Adolescent Health, Murdoch Children’s Research Institute and Royal Children’s Hospital, Melbourne, Australia; Department of Paediatrics, Faculty of Medicine, Dentistry and Health Sciences, The University of Melbourne, Melbourne, Australia; The ALIVE National Centre for Mental Health Research Translation, The University of Melbourne, Melbourne, Australia; Oxford Health NHS Foundation Trust, Oxford, UK; School of Social Sciences, Nottingham Trent University, Nottingham, UK; Department of Psychiatry, Medical Sciences Division, University of Oxford, Oxford, UK; Justice Health Group, enAble Institute, Curtin University, Perth, Australia

**Keywords:** Mental health, schools, health promotion, health promoting schools, comprehensive school health

## Abstract

An independent evaluation of The Resilience Project’s School Partnership Program in Australian secondary schools found that longer participation (6+ years) in this whole-school programme was associated with improved student outcomes, including reduced symptoms of depression and anxiety. This commentary aims to: (a) describe whole-school approaches to improving health and well-being, with reference to their historical context and some selected key studies; (b) highlight the lack of data on the effectiveness of whole-school approaches for reducing depression and anxiety; (c) signal the potential benefits of whole-school approaches when sustainably implemented; and (d) reinforce the need for research that examines links between implementation factors and outcomes. Overall, this commentary underscores the value of viewing schools as complex social systems where multiple components can align to enhance mental health and well-being outcomes for students.

**Figure:**
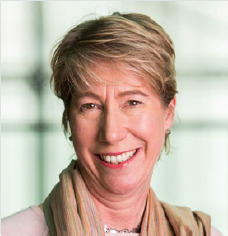

The incidence and impact of common mental disorders such as depression and anxiety in young people demand greater consideration of primary prevention,^1^ including opportunities provided by schools. The majority of school-based approaches to preventing depression and anxiety in young people have focused on the school curriculum as a platform for delivering individually oriented interventions. Many interventions have been adapted from cognitive-behavioural therapy (CBT) and other effective clinical treatments. For example, in a meta-analysis of 137 clinical trials of universal school-based interventions,^2^ 103 were curriculum-based modifications of CBT. These trials were often short in duration, and had null or small effects on student outcomes that were not sustained over time.^2^ In contrast to these approaches, whole-school interventions conceptualise schools as complex social systems consisting of multiple interacting components which can align to amplify benefits for health and well-being, including mental health.^3^ Beyond curricula, these include school health policies, school social and physical environments, links to the wider school community, including parents, and access to health services. In *BJPsych Open*, an evaluation of a programme to prevent depression and anxiety in secondary school students raises some important questions about the effectiveness and implementation of whole-school approaches.

The notion that elements beyond curriculum, teacher–student ratios or teacher competence can influence student academic outcomes, behaviour, health and well-being still feels novel. In the 1970s, an innovative study in 12 secondary schools in London led by the eminent child psychiatrist Professor Sir Michael Rutter found a causal relationship between school processes and students’ academic performance and behaviour. As described in Rutter and colleagues’ subsequent book *Fifteen Thousand Hours* – a reference to the number of hours young people typically spend in schooling – the integration of school processes such as a school’s social organisation and orientation to its students (i.e., academic emphasis, teachers’ actions during lessons, school approaches to rewards and punishments, student responsibilities and participation in the school) and staff organisation (i.e., how staff worked together and how decisions were made within a school) was hypothesised to culminate in daily actions that influenced the ‘ethos’ or social environment of the school.^4^ The authors found that these school processes profoundly influenced student performance and behaviour. In the 1990s, and building on the Ottawa Charter for Health Promotion, the World Health Organization’s framework for Health-promoting Schools helped extend the conceptualisation of schools from learning environments to communities for creating empowered, engaged and healthy students.^3^ Subsequently the first global standards for Health-promoting Schools, published in 2021, more explicitly articulated the importance of schools as a system for achieving education outcomes, health and well-being, and highlighted the importance of sustainable implementation actions.^3^

While a strong evidence base affirms that whole-school approaches can improve many health outcomes such as physical activity and smoking, a long-standing question relates to the ability of whole-school approaches to shift the incidence of depression and anxiety.^5,6^ Several early trials of whole-school approaches consisted of theory-based multi-component interventions focusing on common risk and protective factors within the individual, family, peers and school, rather than specific disorders or behaviours.^7,8,9^ For example, the Seattle Social Development Project targeted primary school classroom management and instruction, children’s social competence, and parenting practices, and achieved many impressive health gains.^10^ Nine years after the intervention, sexual health benefits at age 21 included delayed age of first sexual intercourse, fewer sexual partners and fewer sexually transmitted infections – despite the intervention not addressing any aspect of sexual health.^11^ In terms of mental health, while sustained benefits were found for emotional regulation, there were no long-term effects on anxiety or depression.^10^

These findings are echoed in a recent meta-analysis of whole-school approaches to promoting mental health and reducing risk behaviours.^6^ Disappointingly, only 28 studies were identified, and the authors questioned the extent to which many of these sufficiently involved ‘whole-school’ interventions. Reductions were found for some risk factors for poor mental health, such as cyberbullying, but there were no improvements in depression or anxiety.^6^ An important consideration is how long it can take to implement whole-school approaches, as complex interventions require commitment from school leaders, staff engagement and training, and changes to routine practices, many of which are aided by community partnerships and collaboration between the health and education sectors.

A study by Roshini Balasooriya Lekamge and colleagues^12^ in *BJPsych Open* makes a very specific contribution through its findings relating to depression and anxiety outcomes and the relative differences in student outcomes according to the duration of programme implementation. The authors independently analysed data to assess the effectiveness of The Resilience Project’s School Partnership Program (‘the Program’), an Australian whole-school programme that aims to promote mental health and well-being through a focus on gratitude, empathy, emotional literacy and mindfulness (GEEM). Managed by a community partner, the Program was first implemented in schools over a decade ago and is embedded among government initiatives to promote mental health and well-being in schools. Consistent with Health-promoting Schools, in addition to curriculum-based activities (50 year-level lessons are provided across six years of secondary school), schools are provided with activities to foster GEEM outside the formal curriculum and supportive resources. In addition to reinforcing implementation through professional development for staff, resources are also provided to facilitate the practice of GEEM as a staff cohort and to engage parents and carers who are provided with access to an online information hub consisting of educational videos, family well-being activities and links to additional resources.

The evaluation consisted of a cluster quasi-experimental study of 40 149 students from 102 secondary schools, surveyed in 2023 via an online portal. The intervention arm consisted of students from 55 schools that were actively implementing the Program, while the control arm consisted of students from 47 schools that had never implemented it. Student outcomes were measured at a single time point using validated surveys across five domains (life satisfaction, hope, coping skills, anxiety and depression). Participant findings were stratified by the number of years that their school had implemented the Program (2–3 years, 4–5 years, 6+ years).

The key finding was that longer participation in the Program was accompanied by better student outcomes. While there were no apparent differences in outcomes for students whose schools were in their second or third year of implementation in comparison to the control arm, students from schools that had implemented the Program for 6+ years had better outcomes than control students across all five domains, including depression and anxiety.

The major limitation of this study is that notwithstanding adjustment for differences between intervention and control schools in terms of gender, grade, rurality and socioeconomic status at the time of the survey, schools were not randomised and there were no baseline data. As the authors themselves noted, schools that chose to both engage with the Program and managed to implement it for at least 6 years may have systematically differed from the control schools and from the intervention schools that had only more recently implemented it, especially given the timing and impact of the COVID-19 pandemic. There are multiple avenues through which confounding may have influenced these findings, including school leadership and resourcing.

This finding contributes to a small but growing body of evidence that effects on health outcomes, including mental health outcomes, can be significantly greater the longer an intervention is implemented. This has implications for when to measure the effectiveness of school-based mental health interventions. For example, the SEHER study, a cluster randomised controlled trial of a whole-school health promotion intervention conducted in 75 schools in Bihar, India, found stronger beneficial effects for outcomes such as adolescent depressive symptoms, bullying, violence perpetration and school climate at the 17-month follow-up compared to the 8-month follow-up.^13^ This study built on an earlier Australian whole-school trial, the Gatehouse Project, which aimed to build secondary students’ sense of security and trust, enhance communication and social connectedness with peers and teachers and enhance positive regard though valued participation in diverse aspects of school life.^9^ Four years on, an impressive reduction in a suite of health risk behaviours in intervention schools was found, with a 25% reduction in substance use, antisocial behaviour and early initiation of sexual intercourse.^9^ The Gatehouse Project was actively implemented for 3 years. Interestingly, the observed reductions in ‘any risky behaviours’ and ‘marked risk behaviours’ were significantly greater among a new cohort of Year 8 students who were surveyed four years after the intervention was first implemented when compared to Year 8 students who were surveyed two years after initial implementation, suggesting that the broader school system(s) had been sustainably changed by the intervention.

Any school that is continuing to actively implement a mental health promoting programme beyond six years might reasonably be expected to be doing something right. In the case of the evaluation of GEEM, sustaining the Program’s implementation may have been aided by an active community partner that provided tangible resources and supports which have been shown to influence the sustainability of public health interventions.^14^ Beyond its orientation to students, the Program also focused on the professional development and well-being of teachers, who typically lack pre-service and in-service training around mental health and well-being and whole-school approaches.^14^ The relative benefit of these teacher-specific elements on the effectiveness and sustainability of whole-school mental health programmes remains unknown and warrants more specific evaluation.

The findings of Balasooriya Lekamge and colleagues^12^ suggest that whole-school approaches continue to hold promise for improving mental health outcomes. Research is particularly needed to explore (a) which processes and practices best enhance implementation, especially around changing school socio-emotional environments; and (b) how schools can better integrate curriculum-based interventions aimed at fostering these environments with the wider elements of whole-school approaches to improving mental health. The length of time typically required to shift school systems suggests that particular attention is needed to assess the quality of implementation, with important implications for when to measure the effectiveness of school-based mental health interventions.
